# Salvage of Free Flaps Following Vascular Pedicle Avulsion Using “Supermicrosurgery” Techniques: A Case Report of DIEP Free Flap Salvage and Review of the Literature

**Published:** 2018-03-26

**Authors:** Nicholas Cereceda-Monteoliva, David Izadi, Sherif Wilson

**Affiliations:** Department of Plastic Surgery, Royal Devon & Exeter Hospital, Exeter, United Kingdom

**Keywords:** pedicle injury, avulsion, free flap, supermicrosurgery, salvage

**Figure:**
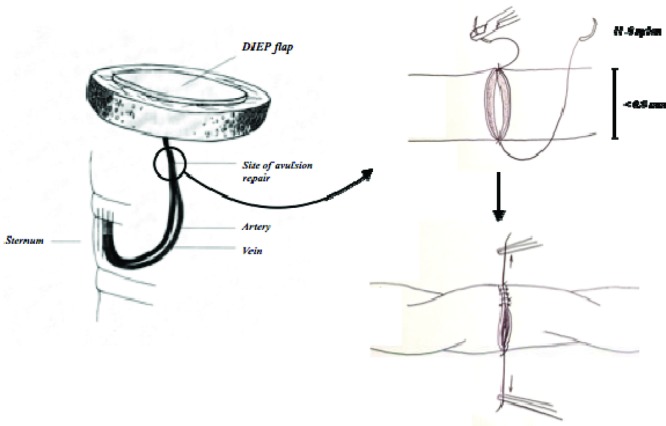


## DESCRIPTION

A 46-year-old woman had a delayed breast reconstruction using a free deep inferior epigastric perforator (DIEP) flap raised on a single medial row deep inferior epigastric perforator, complicated by intraoperative pedicle avulsion. Following a 1-cm excision of the intimally damaged vessel, the pedicle was repaired and the flap was salvaged using a supermicrosurgical technique.

## QUESTIONS

1. What is supermicrosurgery?
2. What levels of pedicle injury can occur?
3. What are the intraoperative strategies to salvage the free flap from pedicle avulsion?
4. What scope is there for future advancements in free flap reconstruction using supermicrosurgery?

## DISCUSSION

Supermicrosurgery was first used in the 1980s, with successful replantation of distal fingertips.^1^ Technological improvements such as advancing microscope optics, finer microsurgical instruments, and suture material and needles, combined with increased operator experience, have equipped surgeons with the skills to perform microvascular dissection and anastomosis of vessels less than 0.8 mm in diameter, setting a new benchmark for microsurgery. Now a whole range of tissues can be used for reconstruction using supermicrosurgery, with widespread applications including lymphatic reconstruction surgery and true perforator-to-perforator free flap surgery.^2^

A vascular pedicle injury is potentially disastrous in free flap surgery. Although uncommon, pedicle injury is an important surgical complication and understanding how it arises is essential to effective management. Different levels of pedicle injury are as follows:

1. *Mishandling* of the pedicle by the surgeon. This can result in intimal injury to an intact vessel.^3^
2. *Avulsion* of the vascular pedicle by excessive traction. Single-vessel avulsion involves either arterial or venous supply to the flap.^4^
3. *Double-vessel avulsion* of both arterial and venous supplies to the flap.^5^
4. *Diathermy injuries* to the pedicle, from direct cautery or local thermal spread.^6^
5. *Anastomotic aneurysmal rupture*, due to a pressure gradient created by mismatched donor and recipient vessels or a poorly constructed anastomosis.^7^

Salvage of free flaps where smaller diameter vessel injury has occurred is now possible using supermicrosurgical techniques. Importantly, the surgeon must excise the zone of trauma prior to repair. Selecting the appropriate operative strategy to then repair the avulsed pedicle will depend on the nature of injury. We therefore propose an algorithm to support this selection:

1. *Before flap inset*: If avulsion occurs while raising the flap, use an alternative perforator if available or repair the injured vessels if none available.
2. *During flap inset*: If avulsion occurs during the flap transplant or microsurgical anastomosis with recipient vessels, reattempt a primary anastomosis. However, if the pedicle is now too short to reach the recipient vessel, use an interposition vein graft to lengthen the vascular pedicle and perform a tension-free anastomosis.^3^
3. *After flap inset*: If avulsion occurs after the flap is inset, identify and perform an anastomosis with another shorter recipient vessel or repair and reanastomose the avulsed vessel.^8^

For *double-vessel avulsion*, repairing the artery first allows the vein to fill and become more prominent for repair. In DIEP breast reconstruction specifically, repairing only the artery produces flap congestion and backpressure that expands the superficial inferior epigastric vein, which can then be used instead.

Free flap vascular pedicle injury remains uncommon but potentially devastating. However, free flap salvage is possible by use of innovative supermicrosurgical techniques and the operative strategies proposed. Supermicrosurgery has already brought a new frontier to free tissue transfer. Autologous transfer of tissue segments for congenital, oncological, and trauma-related defects is now possible. Perforator-to-perforator and free-style flaps, raised on anatomically variable vessels, have increased the diversity of reconstructive options, especially when smaller diameter vessels can be exploited using supermicrosurgical techniques. This will lead to spare-part surgery where disposable tissues with minimal donor morbidity can be used instead of locally destructive reconstructive options. Supermicrosurgical transfer of tissue and organ segments for facial allotransplantation and end-stage organ failure is being explored.^1^ In conclusion, training the next generation of plastic surgeons using supermicrosurgical techniques will improve flap salvage and advance reconstructive practice.
